# Some Points in the Treatment of Scarlet Fever

**Published:** 1911-01-28

**Authors:** A. Knyvett Gordon

**Affiliations:** Formerly Medical Superintendent of Monsall Hospital, and Lecturer on Infectious Diseases in the University of Manchester.


### Hospital Clinics.

### SOME POINTS IN THE TREATMENT OF SCARLET FEVER.

By A. KNYVETT GORDON, M.B.Cantab. Formerly Medical Superintendent of Monsall Hospital, and Lecturer on Infectious Diseases in the University of Manchester.

In this paper I do not purpose entering fully into the treatment of all phases and all varieties of scarlet fever, but shall rather take certain points which I have come to regard as essential in the management of patients suffering from this rather protean disease. Before doing so, however, it will be necessary to touch briefly on the pathology of the infection.

### Two Types of Scarlet Fever.

When a patient becomes infected with the virus of scarlet fever, one of two rather different things may happen: the organisms may lodge either in the fauces or in the circulating blood.

I have purposely put this somewhat dogmatically because I wish to emphasise a distinction which I believe to be of great value clinically, and one, moreover, which has not received the attention in most text-books of medicine to which it is, in my view, entitled. We have really two distinct types of disease.

In some cases the onset is quite sudden, and the patient is acutely ill from the first He is drowsy, and soon becomes semi-comatose; there is great prostration, a high temperature, which persists without any remission, and his lips are cyanosed, but he does not complain of sore throat, and on examination, though there may be a little injection of the fauces, there is no definite tonsillitis, and it is obvious that the throat is even less affected than it would be were the patient to be suffering from an ordinary attack of simple scarlatina. Nor does the brunt of the attack fall on the skin, for when the rash appears—and the patient not infrequently dies in the first twenty-four hours without any rash at all—it is scanty, dusky, and ill-defined.

This type of disease was formerly known as malignant scarlet fever, but inasmuch as in some places that term is applied to an attack of any type of more than ordinary severity, it is better to call it toxic scarlet fever. Until recently the exact pathology of this type was wrapped in obscurity, but the fact that streptococci can be found in the circulating blood in almost every case where a proper technique is employed has cleared up the difficulty. It is, of course, possible that the site of entrance of the organisms may be in the fauces, but the essential point is that the blood, from the first, contains streptococci and their toxins.

### The Ordinary Type.

Let us now take another and rather more common type. Here the patient need not be very acutely ill at the onset, though the initial vomiting and shivering are usually rather more persistent than in a moderately severe attack of simple scarlatina, but it very soon becomes apparent that the fauces are intensely inflamed, and that necrosis of the tonsils and palate has set in; this may be confined to the superficial epithelium, or it may go on to considerable loss of substance and the parts become covered with a thick leathery-looking slough which is not unlike diphtheritic membrane.

From the fauces infection travels to the nose and nasopharynx, and in many cases thence to the conjunctival sacs and middle ears. Toxins are formed at the site of infection, and are absorbed into the blood in more or less direct proportion to the extent of faucial mucous membrane which is involved in the sloughing process; but streptococci are not found in the blood, though they are present, of course, in numbers in the throat. These cases are best described as septic scarlet fever, though some authorities use the term scarlatina anginosa. In addition to these types we have the ordinary attacks of scarlet fever, in which neither the fauces nor the blood are intensely affected.

### Treatment.

Keeping this distinction in mind, it will be obvious that a very different line of treatment is required for each of the three varieties. In a simple attack it is unnecessary—and indeed inadvisable—to attack the fauces with a strong germicide, but it is essential that they shall be kept clean, and that any toxins formed shall be washed away as far as possible, so we irrigate the throat and nose with a large quantity of a non-irritating fluid. After a prolonged trial of most of the advertised antiseptics I have come to prefer normal saline solution, which is preferably applied from a douche-can placed a very little higher than the patient's head. It has been objected that the routine use of the nasal douche tends to produce otorrhœa, but I have elsewhere shown that, in a long series of cases treated with and without this measure, the incidence of otorrhœa was actually lower when the nose as well as the buccal cavity was freely irrigated, except when an antiseptic solution was employed: then otorrhœa was more frequent, doubtless owing to the rhinitis, which seemed to be unavoidable when any chemical other than sodium chloride was added to the water.

### The Hygiene of the Skin.

Another essential in dealing with an ordinary case of scarlet fever is that the skin shall be kept acting throughout the attack, especially during the pyrexial period, and for this purpose nothing is better than total immersion in a bath of tepid or cool water; this should be done at least twice a day during the acute stage, and once a day in convalescence, and it may advantageously be followed by anointing of the skin with oil if the desquamation is free. It is often advisable to add a little eucalyptus to cover the odour of the olive oil, but this has, unfortunately, resulted in eucalyptus being vaunted as a remedy for scarlet fever, which it certainly is not. It is often difficult to persuade the relatives in private practice that immersion is advisable, as it is apt to be regarded as a somewhat heroic remedy, but there can be no doubt that the incidence of subsequent nephritis is lower when the bath has been regularly employed from the first. It is hardly necessary to add that the bowels should be kept well open throughout the attack.

### Septic and Toxic Cases.

With regard to the toxic cases, it is necessary that the treatment should be prompt, and it consists in the administration in adequate doses of a polyvalent antistreptococcic serum; in fact, nothing else is of any use whatsoever in this very fatal form of the disease. Very often the serum is useless, but now and again it acts like magic. Any reliable brand may be employed, and I have been accustomed to give a dose of 100 c.c. in one injection, which is not repeated, doses of 10 or 20 c.c. having not, in my experience, yielded the same results. Massive infusions of normal saline solution are undoubtedly of some benefit, and for convenience the serum may be added to the first injection and the saline alone repeated at intervals of twelve hours until the patient's condition has materially improved. A convenient way of administering the fluid is by means of a douche-can connected with a Y-shaped tube, each branch of which terminates in a needle of large calibre which is inserted into the subcutaneous tissue in the axilla or under the breasts; about two pints may be given in each injection, and the process should take about half an hour. If the patient is conscious enough to swallow, alcohol may be given freely as well, though stimulation is not of much avail by itself, as is shown by the fact that it was practically the only remedy at a time when these toxic cases were found to be almost invariably fatal. It may be necessary to treat hyperpyrexia by cold or iced packs. Disinfection of the fauces is useless.

In the septic type of case, on the other hand, the indication is to curtail the output of toxins at its source, and therefore attack the fauces with as powerful a germicide as it is possible safely to employ. Obviously it is not advisable to use a strong disinfectant in large quantities, so we do not resort to the douche, though irrigations of normal saline are useful to keep the mouth and fauces clean. Nor is the spray as efficacious as some would have us believe; for one thing, the finely divided solution has but little penetrating power, and we are really simply depositing a small quantity of medicament on the top of a slough or layer of mucus. In practice it is best to use a swab.

### A Note on Drugs.

In my experience the best drug for local application is one of the modern non-toxic "high coefficiency" coal-tar disinfectants, in the form of an emulsion; many of these preparations are soapy solutions of the essential oil, and they are too irritating for internal application, though they are quite suitable for disinfecting walls and utensils. Of the emulsified variety, Izal is a good example, but the practical point is that it should be used undiluted, as it has an anæsthetic action which is lost on dilution, and the solution then becomes very irritating. It should be applied deliberately and firmly, with the throat well exposed in a good light, and the superfluous fluid should be mopped off with a second swab soaked in water or normal saline solution; a mouth gag is sometimes necessary.

### Oral Hygiene.

This process should be repeated once or twice a day until the slough has separated and there are signs of healing on the ulcerated surfaces. Serum seems to be quite useless in the septic type of case, though saline subcutaneous infusions are often useful, especially when diarrhœa sets in or signs of collapse appear. Owing to the condition of the fauces the administration of nourishment is often very difficult, and there are few diseases in which the advantages of skilled nursing are so apparent as in septic scarlet fever. In practice it is generally best to employ the nasal tube from the first, so that the patient comes to recognise that its passage is less painful than swallowing before the fauces have become extensively ulcerated. If its use is deferred to this stage the terror with which its passage is at first associated is apt to put a severe strain on the heart. About four ounces of milk, plain or peptonised, should be given every four hours, and an egg or a little brandy may be added, according to the state of the digestive and circulatory organs.

### Restlessness.

A practical difficulty that arises in the treatment of the septic type of case is the restlessness of the little patients. Now, this is seldom present when the child is able to drink large quantities of fluid, and it is similarly greatly mitigated by the employment of subcutaneous saline infusions; but it is not possible to continue this method of administration *ad infinitum*, and we then have to give water, or preferably thin barley-water, by the nasal tube or per rectum. Unfortunately, however, the latter method is apt to set up diarrhœa, which we particularly wish to avoid. Here again the skilful nurse is invaluable. In practice the prognosis is almost always good when we can give water in one way or another, and in my opinion the majority of fatal cases really succumb to a deficiency of fluid; probably the remedy acts by washing out accumulated toxins. Sometimes we can do good by assisting the action of the skin with a hot pack or a radiant heat bath, which can be easily extemporised by suspending a couple of incandescent lamps from a cradle placed over the patient as he lies in bed.

In this type of case otorrhœa is very common and nephritis not rare; but the treatment of these sequelæ, though most important, does not fall within the scope of the present paper.

